# Molecular Classification of Endometrial Stromal Sarcomas Using RNA Sequencing Defines Nosological and Prognostic Subgroups with Different Natural History

**DOI:** 10.3390/cancers12092604

**Published:** 2020-09-11

**Authors:** Mehdi Brahmi, Tatiana Franceschi, Isabelle Treilleux, Daniel Pissaloux, Isabelle Ray-Coquard, Armelle Dufresne, Helene Vanacker, Melodie Carbonnaux, Pierre Meeus, Marie-Pierre Sunyach, Amine Bouhamama, Marie Karanian, Alexandra Meurgey, Jean-Yves Blay, Franck Tirode

**Affiliations:** 1 Department of Medical Oncology, Centre Léon Bérard, 28 rue Laennec, 69008 Lyon, France; isabelle.ray-coquard@lyon.unicancer.fr (I.R.-C.); armelle.dufresne@lyon.unicancer.fr (A.D.); helene.vanacker@lyon.unicancer.fr (H.V.); melodie.carbonnaux@lyon.unicancer.fr (M.C.); jean-yves.blay@lyon.unicancer.fr (J.-Y.B.); 2 Cancer Research Center of Lyon, Centre Léon Bérard, Univ Lyon, Claude Bernard University Lyon 1, INSERM 1052, CNRS 5286, 69008 Lyon, France; daniel.pissaloux@lyon.unicancer.fr (D.P.); marie.karanian@lyon.unicancer.fr (M.K.); 3 Department of Biopathology, Centre Léon Bérard, 28 rue Laennec, 69008 Lyon, France; tatiana.franceschi@lyon.unicancer.fr (T.F.); isabelle.treilleux@lyon.unicancer.fr (I.T.); alexandra.meurgey@lyon.unicancer.fr (A.M.); 4 Department of Surgery, Centre Léon Bérard, 28 rue Laennec, 69008 Lyon, France; pierre.meeus@lyon.unicancer.fr; 5 Department of Radiation Oncology, Centre Léon Bérard, 28 rue Laennec, 69008 Lyon, France; marie-pierre.sunyach@lyon.unicancer.fr; 6 Department of Radiology, Centre Léon Bérard, 28 rue Laennec, 69008 Lyon, France; amine.bouhamama@lyon.unicancer.fr

**Keywords:** endometrial stromal sarcomas, RNA-sequencing, gene fusions, prognostic, uterine, sarcoma, expression profile

## Abstract

**Simple Summary:**

In about half of the cases, endometrial stromal sarcomas lack the canonical oncogenic fusions *JAZF1-SUZ12* or *YWHAE-NUTM2*, which are mutually exclusive. The aim of this study was to explore by RNA sequencing a retrospective series of uterine sarcomas diagnosed as endometrial stromal sarcomas but negative for *JAZF1* and/or *YWHAE* rearrangement in FISH, in order to provide a better description of their molecular landscape, improve the classification of endometrial stromal sarcomas and provide guidance for disease management.

**Abstract:**

A series of 42 patient tumors diagnosed as endometrial stromal sarcoma (ESS) based on the morphology but negative for *JAZF1* and/or *YWHAE* rearrangement in FISH was analyzed by RNA-sequencing. A chromosomal rearrangement was identified in 31 (74%) of the cases and a missense mutation in known oncogenes/tumor suppressor genes in 11 (26%). Cluster analyses on the expression profiles from this series together with a control cohort composed of five samples of low grade ESS harboring a *JAZF1-SUZ12* fusion, one high grade ESS harboring a *BCOR*-ITD, two uterine tumors resembling ovarian sex cord tumors, two samples each of uterine leiomyoma and leiomyosarcomas and a series of *BCOR*-rearranged family of tumor (*n* = 8) indicated that tumors could be gather in three distinct subgroups: one mainly composed of *BCOR*-rearranged samples that contained seven ESS samples, one mainly composed of *JAZF1*-fused ESS (*n* = 15) and the last composed of various molecular subtypes (*n* = 19). These three subgroups display different gene signatures, different in silico cell cycle scores and very different clinical presentations, natural history and survival (log-rank test, *p* = 0.004). While *YWHAE-NUTM2* fusion genes may be present in both high and low grade ESS, the high-grade presents with additional *BCOR* or *BCORL1* gene mutations. RNAseq brings clinically relevant molecular classification, enabling the reclassification of diseases and the guidance of therapeutic strategy.

## 1. Introduction

Endometrial stromal sarcoma (ESS) is a rare disease accounting for less than 1% of all uterine tumors [1]. It represents the second most common type of uterine mesenchymal neoplasm, after leiomyosarcoma (LMS). ESS gathers a heterogeneous group of tumors with distinct clinical, histological and genetic characteristics. According to the 2014 WHO (World Health Organization) Classification [2], ESS is divided into three categories: low-grade ESS (LG-ESS), high-grade ESS (HG-ESS) and undifferentiated uterine sarcoma (UUS).

In addition to morphological and immunohistochemical data, the integration of molecular methods enables a more precise diagnosis and nosological classification. It may be important for guiding clinical management in entities with different natural histories. The current state of the art indicates that ESS is characterized by different types of recurrent genetic events, most commonly chromosomal translocations that frequently involve chromatin remodeling genes. The two most frequent gene fusions are *JAZF1-SUZ12* [3], known to be associated with LG-ESS and favorable prognosis, and *YWHAE-NUTM2* [4], known to be related to HG-ESS and high risk of rapid relapse and death. Adjuvant therapy is not recommended for *JAZF1*-fused LG-ESS, and in the case of advanced disease, aromatase-inhibitors (AIs) are the standard treatment [5]. Conversely, *YWHAE*-fused HG-ESS are considered as high risk tumors for which adjuvant chemotherapy and radiotherapy is often recommended, and in the case of advanced disease, they are considered to be insensitive to hormonal therapies, and cytotoxic chemotherapy is considered appropriate [6,7].

Grading and molecular classification are therefore of importance to classify ESS and decide on a treatment. In current routine practice, *JAZF1-SUZ12* and *YWHAE-NUTM2* fusion genes are screened by fluorescence in situ hybridization (FISH), which has limitations: (i) the most commonly used FISH test being a break-apart probe allows detection of only one of the rearranged genes and may induce false negatives; (ii) about half of ESS does not harbor a fusion of *JAZF1* or *YWHAE*. Involvement of the *PHF1* gene in chromosomal rearrangements targeting band 6p21 has been found in LG-ESS with different partners from JAZF1, EPC1, MEAF6 and BRD8 [8,9,10], and some other recurrent rearrangements have been reported involving *BCOR* [11,12,13], *NCOA* [14,15] or *MBTD1* [16]. Recently, the expression of BCOR using IHC (Immunohistochemistry) was proposed as a criterion to classify HG-ESS carrying a *BCOR* rearrangement or a *YWHAE-NUTM2* fusion [17].

In order to improve ESS classification and provide guidance for disease management, we characterized the molecular alteration of LG- and HG-ESS lacking canonical translocations, investigating a retrospective series of “FISH-negative” ESS using RNA-sequencing. We hypothesized that not only RNA sequencing would help in a better risk stratification of ESS but would also provide a better description of their molecular landscape by gathering their genetic abnormalities and specific expression profiles.

## 2. Results

### 2.1. Clinical and Pathological Features

This translational study was conducted on a retrospective series of 42 ESS negative for *JAZF1* rearrangement (*n* = 22), *YWHAE* rearrangement (*n* = 11) or both (*n* = 9) (Figure S1). Patients and tumors characteristics are presented in Table 1 and Table S1.

Using IHC, 62% (*n* = 26) demonstrated a nuclear positivity of ER (Estrogen receptors) and/or PR (Progesterone receptors) and 76% (*n* = 32) were positive for CD10. Overall, 26 (62%) were considered as LG-ESS using simple morphological classification including 4 (10%) with sex cord-like elements, 7 (16%) as HG-ESS and 8 (19%) as undifferentiated uterine sarcomas (UUS); one (2%) could not be graded. Median age at diagnosis was 53 years (26 to 82 years). Thirty-nine (93%) were located in the uterus (two primary ESS of the ovary and one of the peritoneum). Twelve patients (29%) were metastatic at diagnosis (FIGO stage IV), four LG, three HG and five UUS. The median overall survival (mOS) of the whole cohort was 11.3 years. For FIGO stage IV disease, treatments received by patients (and duration) are detailed in Table S2.

### 2.2. Molecular Classification Using RNA-Sequencing

**Figure 1:**
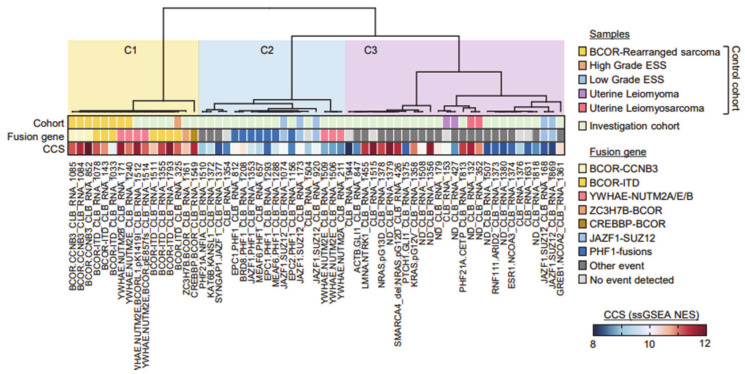
Consensus hierarchical unsupervised clustering of expression profiles extracted from FFPE tumor samples. Consensus for k = 3 cluster is shown. Sample, tumor types and identified fusion genes are indicated. Single sample GSEA (Gene Set Enrichment Analysis) G2/M cell cycle score (CCS) for each samples is represented in a blue–red normalized scale.

All but one RNA-sequencing successfully passed the quality controls (98%, *n* = 41). A chromosomal rearrangement was identified in 31 (74%) samples. The detected gene alterations are listed in Table S3. Briefly, an *YWHAE-NUTM2E* gene fusion was identified in 5 cases (12%), 10 samples (24%) carried a fusion involving a gene of the PHD family (*PHF1*, *PHF21A* or *KAT6B*), *BCOR*-rearrangements were observed in 5 (12%) samples, including 3 *BCOR* internal tandem duplications (*BCOR*-ITD), one *ZC3H7B-BCOR* fusion and a novel fusion, *CREBBP-BCOR* (Figure S2), and two (5%) samples carried a fusion involving genes of the *NCOA* family. In a single sample each, a *SYNGAP1-JAZF1* and a new gene fusion involving a chromatin remodeling gene (*RNF111-ARID2*) were identified. In nine remaining samples, fusion genes could be identified but were either numerous, unrelated to the chromatin remodeling process and of unknown function or described in other neoplasia than ESS. Among the latter, two cases (5%) harbored a fusion implicating *GLI1* relating them to the previously described “malignant epithelioid neoplasm with *GLI1* gene rearrangements”, and one case (2%) harbored a *LMNA-NTRK1* fusion corresponding to the previously described *NTRK*-fused uterine sarcomas [18]. Finally, in eight samples (19%) we could not detect any in-frame fusion gene. Of note, five (11%) cases harbored complex genomic profiles with numerous gene fusions and/or had mutations involving either *TP53*, *KRAS*, *NRAS* or *BRAF*, consistent with a diagnosis of UUS, and one case presented a deletion of *SMARCA4* (together with a *NRAS* mutation), consistent with a *SMARCA4*-deficient undifferentiated uterine sarcoma. Of note, any time a rearrangement involving *PHF1*, *YWHAE* or *JAZF1* was revealed by the RNAseq, a FISH (*PHF1* or *YWHAE* or *JAZF1*) was performed in addition. The expression profiles of this series was matched to a control cohort of 20 recently diagnosed cases, including 5 samples of LG-ESS harboring a *JAZF1-SUZ12* fusion, 1 HG-ESS harboring a *BCOR*-ITD, 2 uterine tumors resembling ovarian sex cord tumors (UTROSCT) and 2 samples each of uterine leiomyoma and LMS. Because of the similarity of their genetic alterations, we also included eight samples of soft-tissues and bone sarcomas carrying a *BCOR*-rearrangement (two pediatric undifferentiated round cell soft tissue sarcomas (*BCOR*-ITD), two pediatric undifferentiated round cell bone sarcomas (*BCOR-CCNB3*) and one pediatric brain tumor (*BCOR*-ITD)] or an *YWHAE-NUTM2* fusion (two pediatric undifferentiated round cell bone sarcomas). Unsupervised consensus hierarchical clustering divided the samples into three subgroups (Figure 1 and Figure S3).

Cluster C1 contained all the *BCOR*-rearranged sarcomas, and four samples carried a *YWHAE-NUTM2* fusion (two ESS and two from the control cohort). Cluster C2 was composed of most *JAZF1*-fused and all *PHF1*-fused ESSs from both the control and investigation cohorts, together with three *YWHAE-NUTM2* positive ESS samples. Cluster C3 grouped various molecular subtypes, including the *NCOA*-fused ESS, close to the cases of uterine LMS from the control cohort, and contained all the samples consistent with UUS.

**Figure 2:**
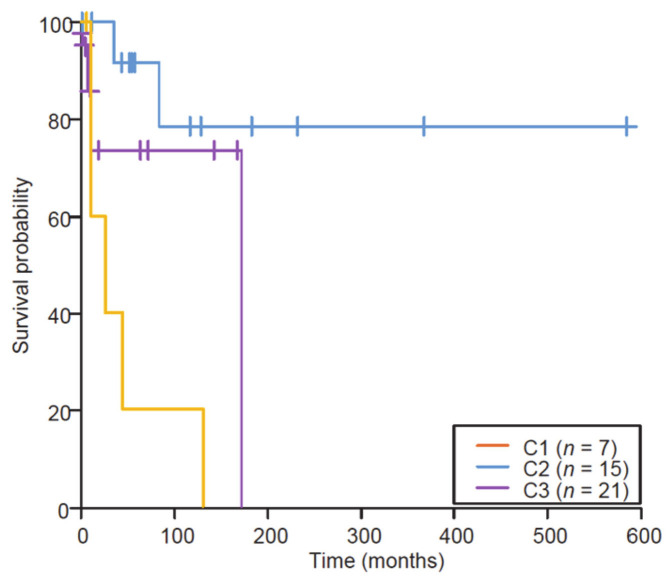
Kaplan–Meier survival analysis of the retrospective series of primary diagnosed ESS stratified by the clusters (log-rank, *p* = 0.004; Wilcoxon); x-axis, time in months. The three subgroups display different gene signatures and different in silico cell cycle scores that significantly correlated with different overall survivals.

Evaluation of a cell cycle score (CCS), based on single sample GeneSet Enrichment Analyses (ssGSEA) on the hallmark G2/M MsigDB geneset, showed that C1 samples had a high CCS, C2 samples had a rather low CCS and the C3 cluster was mixed. These clustering data were significantly correlated to survival (Figure 2; log-rank test, *p* = 0.004).

Indeed, ESSs from the C1 group (high grade; HG) showed a more aggressive clinical behavior (mOS reached at 18 months), while ESSs from the C2 group (low grade; LG) were much more clinically indolent (mOS not reached), and C3 (intermediate grade; IG) showed clinically intermediate behaviors (mOS not reached). Amongst 12 cases that had synchronous metastasis, 3 clustered in the C1/HG group (43%, *n* = 3/7), and 8 clustered in the C3/IG group (38%, *n* = 8/21), but only one clustered in the C2/LG group (7%, *n* = 1/15), C2 tumors being less frequently metastatic at diagnosis (Fisher’s exact test, *p* = 0.03).

Finally, the proportion of ER and/or PR positive cases according to the cluster analysis was 2/7 (29%) in C1/HG, 14/14 (100%) in C2/LG and 12/21 (57%) in C3/IG (Fisher’s exact test, *p* = 0.0009).

Overall, as shown in Figure S4, the alteration detection coupled to expression profile clustering enabled reclassification of 12 cases (29%). Seven cases diagnosed as LG-ESS were reclassified either as HG-ESS with *BCOR* rearrangement (*n* = 2), malignant epithelioid neoplasm with *GLI1* rearrangement (*n* = 2), *NCOA* fusion-positive uterine tumors (*n* = 2) or uterine leiomyoma (*n* = 1). Two cases diagnosed as HG-ESS were reclassified as UUS, and three cases diagnosed as UUS were reclassified either as HG-ESS (*n* = 1), *NTRK* fusion-positive uterine sarcomas (*n* = 1) or *SMARCA4*-deficient uterine sarcoma (*n* = 1). Finally, one case diagnosed as ESS without the possibility of gradation was classified as LG-ESS.

### 2.3. Differentially Expressed Genes and Pathways

Supervised analyses highlighted genes specifically expressed in the defined clusters (Figure S5A–E and Table S4). We identified a significant difference regarding genes involved in the cell cycle/proliferation pathways, morphogenesis/development, translation and spliceosome (upregulated in the C1 as compared to the C2 and C3) and genes involved in immunity (downregulated in the C1 as compared to the C2 and C3). Comparing C2 and C3, we also identified significant differences regarding genes involved in the cell cycle, inflammation and immunity (down-regulated in C2 as compared to C3) and regarding genes involved in neuron/synapse and translation (up-regulated in C2 as compared to C3).

We noticed that the *WT1* gene was often absent or with low expression levels in the C1 tumors as compared as the C2/3 tumors (Figure S4E), and the level of expression of *ESR1* was also significantly higher in the C2/3 than in the C1.

### 2.4. YWAHE-NUTM2B Fusion Are Not Uncommon in LG-ESS

Interestingly, the five cases harboring the *YWAHE-NUTM2B* fusion were divided by the clustering: two cases clustered as expected in the C1 group (HG), whereas three cases clustered in the C2 group (LG), with pure low-grade morphological features (Figure S6). To rule out the fusion gene being expressed by a small number of high-grade cells within low-grade cells, we first looked at the normalized number of reads covering the fusion point without finding differences between the HG and LG samples (around one supporting read per million of mapped reads in both groups). Second, we found at least 80% of the cells having the *YWHAE* rearrangement in FISH analyses. However, the analysis of the two HG showed two nucleotide variation events identified: one mutation in *BCOR* (CLB_RNA_1514: NM_017745: exon4:c.2570_2571del:p.E857fs; AAR = 0.28, validated by Sanger sequencing) (Figure S7), and one in *BCORL1* (CLB_RNA_1512: NM_021946: exon7:c.A4256T:p.K1419I; AAR = 0.67).

The analysis of the differentially expressed genes/pathways between the two groups’ *YWHAE-NUTM2B*-postive cases (Figure S8 and Table S5) identified upregulated genes involved in cell cycle/proliferation pathways and mitochondria in the HG cases, while genes involved in immune response, inflammation, angiogenesis and the extracellular matrix were found downregulated in the HG cases. All LG *YWHAE-NUTM2B*-postive cases expressed high levels of *ESR1*, while it was not expressed in the HG cases (Figure S8C), a result that was further confirmed by IHC.

We then looked specifically to the medical records of those five patients. All three patients in the C2 group were alive without disease (21, 12 and 7 years after the diagnosis). Conversely both patients of the C1 group died of the disease 10 months after the diagnosis for the patient with initial stage 4 and 12 years after diagnosis (for the second patient who had a late relapse at 10 years).

### 2.5. BCOR Rearrangements Are Associated with a Poor Outcome

Unlike *YWHAE-NUTM2* positive cases, all the *BCOR*-rearranged tumors clustered in the C1 group. Among the five cases of our series, we had clinical data for three patients who had all died of the disease, 10 months, 2 years and 3 years, respectively, after the diagnosis.

### 2.6. Immune Landscape

**Figure 3:**
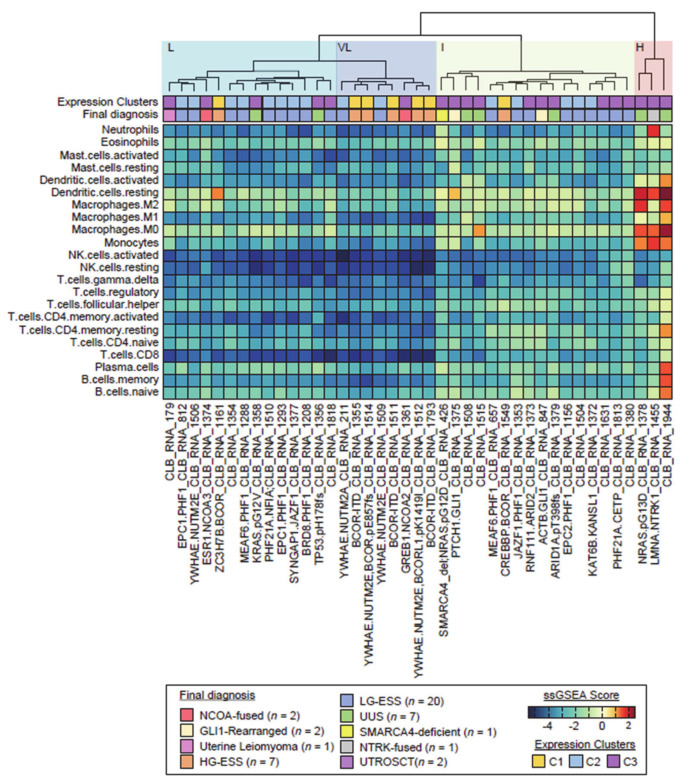
Hierarchical unsupervised clustering of the investigation cohort (*n* = 43) upon immune infiltrate population profiles. Cluster analyses generated one group with high (H) immune infiltrate and one group with low immune infiltrate subdivided in 3 slightly different subtypes: very low (VL), low (L) and intermediate (I). Clusters from Figure 1 and final diagnosis are also reported.

The 41 ESS samples were classified according to immune infiltrate: four main different groups were identified by cluster analysis (Figure 3).

Importantly, a small subset of tumors (composed of a case of UUS that harbored a *NRAS* G13D mutation, a case of uterine sarcoma with *LMNA-NTRK1* fusion and a case of UUS without relevant genomic alteration) stood out consistently from the other tumors. Those three tumors were highly infiltrated, mostly by macrophage immune cell types (subgroup H; high immune infiltrate). The other 38 tumors had limited or no immune cell infiltrates. Nevertheless, three slightly different subgroups could be distinguished among those tumors: VL (very low immune infiltrate), L (low immune infiltrate) and I (intermediate). The VL subgroup (*n* = 8; including four *YWHAE-NUTM2* fusion, three *BCOR-ITD* and the *GREB1-NCOA2* positive tumors) was characterized by a very low immune infiltrate for all cell types, while the L subgroup (*n* = 14; including most *PHF1*-fused tumors) harbored higher densities of macrophages, and the I subgroup I (*n* = 16; including 3 UUS and the two *GLI1*-rearranged neoplasm) was characterized by higher densities of CD4+ T cells and macrophages.

## 3. Discussion

In the present work, we analyzed ESS lacking the canonical fusion genes and described a novel classification of these rare sarcomas, which provides guidance to predict their prognosis and to guide treatments. ESS includes indeed a variety of different disease types with different genomic alterations and natural histories. RNA-sequencing identified cytogenetically negative ESS and enabled us to distinguish three biologically homogeneous risk groups by combining fusion gene discovery and expression profiling. The HG group is composed of ESS harboring a *BCOR* rearrangement, while the LG group is composed of ESS that harbors a fusion of a *PRC2* zinc finger protein (e.g., JAZF1, PHF1). In addition, a third group considered as the IG group, close to the LMS of the control cohort, was composed of various molecular subtypes including *NTRK* fusion, *SMARCA4* loss, *NCOA* fusion and other genetic abnormalities such as *NRAS*/*KRAS* mutations. Importantly, we showed that *YWHAE-NUTM2* tumors are split in the HG and LG group.

Currently, the natural history prognosis and standard treatment of ESS depend on the histological grade of the tumor. According to the current WHO classification [19], *YWHAE*-fused ESS represents a clinically aggressive subtype of ESS classified as HG-ESS, distinct from the usual LG-ESS with *JAZF1* rearrangement and from UUS with no identifiable molecular aberration. In detail, standard local treatment of LG-ESS is “en bloc” total hysterectomy (including bilateral salpingo-oophorectomy), and in cases of advanced disease, AIs are usually preferred. In the case of HG-ESS and UUS, previous clinical studies have shown that adjuvant radiotherapy and adjuvant chemotherapy might improve overall survival [20], and in cases of advanced disease, conventional systemic chemotherapy is recommended [7]. Considering the molecular classification using RNA sequencing, the LG group has a very good prognosis, and adjuvant therapy should not be considered while in case of advanced disease, AIs should remain the standard. Conversely, the HG group has a very poor prognosis, and as they grouped together with the *BCOR–CCNB3* positive samples (so called “Ewing-like” sarcomas) from the control cohort, describing a very-well defined cluster, the therapeutic strategy should be discussed, both in the adjuvant and the metastatic setting, with potentially a more intensive multiagent chemotherapy regimen. The IG group shows an intermediate behavior, the role of AIs in advanced disease is much more uncertain than in the LG group, and RNAseq is crucial as it could guide the treatment (e.g., *NTRK*-fused uterine sarcomas). Finally, RNAseq is essential for the *YWHAE-NUTM2* positive ESS in order to split the tumors in HG/LG and guide the therapeutic strategy.

The results presented here show that molecular analysis is therefore important to assess the prognosis and guide the clinical management. Indeed, 50% of ESSs do not harbor a *JAZF1* and *YWHAE* rearrangement by FISH, which represents an insufficient examination for the characterization of these cancers. Given the heterogeneity of these cancers and the frequent absence of canonical mutations, RNAseq represents an efficient diagnostic tool in routine to identify accurately the different subtypes of ESS with completely opposite natural history and prognosis. Importantly, RNAseq also enabled the reclassification of a quarter of the ESS, such as a tumor initially classified as LG-ESS and finally corresponding to a *ZC3H7B-BCOR* HG-ESS (CLB_RNA_1161) and a tumor initially classified as HG-ESS and finally harboring a *LMNA-NTRK* fusion (CLB_RNA_1455). The first patient presented with a stage IV FIGO disease at diagnosis. She had a 90 mm uterine tumor, microscopically composed of ovoid cells with a myxoid background, low mitotic count, no tumor necrosis and no vascular invasion, strongly positive for CD10 and Cyclin D1, weakly positive for hormone receptors. She received an AI as a first line treatment. Unfortunately, she progressed rapidly and died in less than six months. With the benefit of hindsight, those *ZC3H7B-BCOR* ESSs are indeed known to be associated with abundant myxoid stroma and with aggressive clinical behavior. The second patient had a rapidly progressive disease after an anthracycline-based chemotherapy regimen and did not have time to enter an NTRK inhibitor trial or routine treatment. Similarly, UUS reclassified as *SMARCA4*-deficient uterine sarcoma (CLB_RNA_426), also called “malignant rhabdoid tumor of the uterus”, is a candidate to anti-PD1 in a clinical trial given the high response rates to immunotherapy in this entity (ClinicalTrials.gov Identifier: NCT03012620). In addition, a case initially diagnosed as LG-ESS was reclassified as leiomyoma (CLB_RNA_179). Microscopically, the tumor cells were strongly positive for Desmin, Cyclin D1 and WT1, moderately positive for ER/PR, weakly positive for SMA (smooth muscle actin) and negative for CD 10, and the expert pathologist concluded it was “ESS with smooth muscle cells differentiation”. It is well known that, by histopathological examination and IHC, the distinction between ESS with smooth muscle cell differentiation and highly cellular leiomyomas is challenging [21,22]. At any rate, RNAseq and clustering data helped us correct this diagnosis.

While Sumathi et al. [21] previously showed that diffuse WT-1 positivity is characteristic of endometrial stromal neoplasms and may be of value in diagnosis, we noticed that the WT1 gene is often absent or with low expression levels in HG tumors as compared to the others (Figure S4E), suggesting that WT-1 might be a prognosis factor in ESS. Of note, the level of expression of ESR1 was also significantly higher in the C2/3 than in the C1 cluster (HG), which is consistent with the known prognosis of ER in sarcomas [23]. Importantly, the *YWHAE-NUTM2* fusion gene does not seem to be specific to HG-ESS. Aisagbonhi et al. [24] previously described a case of ESS with a typical morphology of low-grade (without high-grade areas) and a *YWHAE* rearrangement. Here, we demonstrate that (i) LG-ESS with *YWHAE-NUTM2* fusions are not uncommon; (ii) they differ from their HG counterparts not only by the expression of genes involved in cell cycle/proliferation pathways and absence of mutation of the BCOR pathway, but also by those involved in the immune response or angiogenesis and (iii) they may be identified by ER positivity in IHC. Investigation of a larger cohort may confirm these conclusions. Expression data also identified some potentially new therapeutic targets, such as NTRK3 and the histone–lysine N-methyltransferase enzyme, EZH2, which has a higher expression in C1 cluster samples. Inhibition of HDAC (Histone deacetylases) therefore provides a very interesting approach to treatment for this subtype of sarcoma. Preclinical studies have previously shown that ESS may be sensitive to HDAC inhibition, and clinical data of the phase II trial of panobinostat in advanced sarcoma are consistent [25], even if only three ESSs were enrolled in the study. Moreover, a recent paper suggested that ESS with BCOR-rearrangement harbors *MDM2* amplifications [26]. Among the four HG-ESS-BCOR of our series, only one (CLB_RNA_1549) carries a *MDM2-CDK4* amplification (Figure S9).

Finally, while cases of the HG group show very low immune infiltrate for all tumor microenvironment cell types, some of the LG and IG group display moderate immune infiltrates with especially a significant presence of activated T cells (CD3+ CD8+) and regulatory T cells that might be of interest for immunotherapy approaches [27]. Of note, a trial of Pembrolizumab in rare subtypes of sarcomas is ongoing but included only few ESS (ClinicalTrials.gov Identifier: NCT03012620).

By showing the molecular landscape of fusions genes in ESS, RNAseq analysis also helps in understanding the biology and the mechanisms of tumorigenesis. The common altered genes identified in ESS are involved in chromatin remodeling, as SUZ12 belongs to the PRC2 complex while PHF1 promotes the recruitment of PRC2 to the chromatin. Polycomb Repressive Complexes (PRC) 1 and 2 are responsible for silencing the chromatin via histone methylation and ubiquitination. The *JAZF1-SUZ12* fusion disrupts the PRC2 complex, preventing its binding, and thus impairs the chromatin repression of oncogenes such as *HOXA9* [28]. BCOR is part of the non-canonical PRC1.1 complex that binds to BCL6 and acts as a corepressor. The precise mechanisms underlying the different *BCOR*-rearrangements are still unknown, but they possibly disrupt PRC1.1 complex formation [29]. The *SYNGAP1-JAZF1* fusion may be linked to *JAZF1-PHF1* fusion, as both partners are adjacent genes and that a *PHF1-SYNGAP1* is a known readthrough. The *CREBBP-BCOR* fusion may be functionally related to the *CREBBP-BCORL1* recently described in two cases of ossifying fibromyxoid tumors [16], a tumor type that shares fusion genes similar to those of ESS [30]. In three cases of “JAZF1-negative” ESS, we identified other genetic abnormalities involving genes related to chromatin remodeling. In one case (CLB_RNA_1818), we detected a single mutation in the Tet methyl cytosine dioxygenase 2 (*TET2*) gene. TET2 increases the activity of HDAC2 [31], and mutations of this gene have previously been described in other neoplasms such as AML (acute myeloid leukemia) with an unfavorable prognostic value [32]. In one case (CLB_RNA_1372), we detected a *KAT6B-KANSL1* fusion that was previously described in two cases of leiomyomas [33,34]. *KAT6B* encodes a lysine acetyltransferase involved in histone tail modifications. A *RNF111-ARID2* fusion was identified in one patient (CLB_RNA_1373). ARID2 is a subunit of the PBAF chromatin-remodeling complex, and mutations of this gene have been described in various neoplasms (hepatocellular carcinoma, melanoma). Altogether and amongst all tumors that we finally classified as ESS (see Table S3), only five samples remained without having any detectable alteration of a chromatin remodeling gene.

As a limitation of our study, it is important to mention that our study cohort included only five cases of *YWHAE-NUTM2* positive ESS. In addition, without paired normal samples, we do not know whether the variations identified by RNAseq are somatic or even driver mutations. The few variations we reported here are not described in normal tissues and most are present in the COSMIC database [35]. Even so, we cannot definitely rule out private or germline variations, and it will require germline DNA to ascertain this point. Finally, our study did not include any validation cohort, and future studies of larger cohorts of uterine stromal sarcomas are needed to confirm and to expand upon our results and are currently ongoing in our center.

## 4. Materials and Methods

All patients signed informed consent to participate to research according to the French laws. Of note, an Institutional Review Board (The Centre Léon Bérard Clinical Trial Review Committee, Ethical code: N° CMT-2016-013) reviewed and agreed with the study protocol and the informed consent form on the 24th March 2016. The histological diagnosis and grading was established by an expert gynecologic pathologist according to the World Health Organization (WHO) Classification of Tumors [16]. Detailed methods are presented in the Supplementary Materials.

## 5. Conclusions

To our knowledge, our study represents the largest series of RNA-sequenced uterine stromal sarcomas. Altogether, ESSs represent a heterogeneous group of tumors, in terms of clinical behavior, pathology, genetics and immune infiltrate. FISH negative uterine stromal sarcomas present new and recurrent molecular variants of ESS, some of which are previously unreported. RNAseq also allowed the reclassification of a quarter of the tumors and helped in the identification of ESS with opposite natural history and prognosis, which is crucial for treatment decision. The functional similarities between the classical *JAZF1-SUZ12* and the alternative transcripts suggest they activate an analogous oncogenic pathway. We also show that while *BCOR*-rearrangements seem always related to HG and poor prognosis, the detection of *YWHAE-NUTM2B* fusion is not sufficient to consider tumors as HG. Finally, RNA-seq is a powerful tool to enable nosological classification, identify target genes, predict natural history and guide treatment for patients with uterine sarcomas.

## Figures and Tables

**Table 1:** Clinical and pathological characteristics of the investigational series of endometrial stromal sarcoma (ESS).

Characteristics	*N* = 42
**Median Age (years); range**	53; 26–82
**Median size of the primary tumor (mm)**	90
**Pathological diagnosis (review)**	
Low grade ESS	26
(Sex cord elements)	(4)
High grade ESS	7
Undifferentiated uterine sarcoma	8
No possibility of gradation *	1
**FISH results**	
JAZF1 negative (YWHAE not performed)	22
YWHAE negative (JAZF1 not performed)	11
Both JAZF1 and YWHAE negative	9
**Stage at diagnosis**	
Localized	30
Metastatic	12

* One case initially diagnosed as “ESS with indefinite grade” because of unusual features for a low grade sarcoma (tumor necrosis, epithelioid cells, high cyclin D1 expression).

## Supplementary Materials

The following are available online at https://www.mdpi.com/2072-6694/12/9/2604/s1, Figure S1: CONSORT diagram of investigational and control cohorts, Figure S2: Schematic of the previously unreported fusion gene *CREBBP*-*BCOR*, Figure S3: Consensus hierarchical unsupervised clustering of the investigation cohort only, Figure S4: Tumor reclassifications, Figure S5: Supervised analyses between clusters from Figure 1, Figure S6: Photomicrograph shows sheets or fascicles of ovoid to spindle cells with low mitotic count and no tumor necrosis, Figure S7: Schematic of the BCOR frameshift mutation in the CLB_RNA_1514 sample (HG YWHAE-NUTM2B), Figure S8: Supervised analyses between clusters 1 and 2 of the *YWHAE-NUTM2*-positive ESS samples, Figure S9: CGH array analysis of the CLB_RNA_1661 sample (HG ZC3H7B-BCOR), Table S1: Patients and tumors characteristics, Table S2: Systemic treatments received by patients in case of metastatic disease and duration of response (Progression free survival in months), Table S3: Details of the RNAseq fusion and mutation detection, Table S4: Differentially expressed genes between clusters, Table S5: Differentially expressed genes between high grade (C1 cluster) and low grade (C2 cluster) YWHAE-NUTM2-positive ESS.
